# Global research trends in non-muscle invasive bladder cancer: Bibliometric and visualized analysis

**DOI:** 10.3389/fonc.2022.1044830

**Published:** 2022-11-17

**Authors:** Sheng Deng, Fanchao Meng, Lu Wang, Zhen Yang, Lihua Xuan, Zhihua Xuan, Jisheng Wang

**Affiliations:** ^1^ Department of Andrology, Shunyi Hospital, Beijing Hospital of Traditional Chinese Medicine, Beijing, China; ^2^ Department of Urology Surgery, The Third Affiliated Hospital of Beijing University of Chinese Medicine, Beijing, China; ^3^ Department of Surgery, Beijing Xuanwu Traditional Chinese Medicine Hospital, Beijing, China; ^4^ Department of Andrology, Dongzhimen Hospital, Beijing University of Chinese Medicine, Beijing, China

**Keywords:** non-muscle invasive bladder cancer, bibliometrics, data visualization, research status, hotspots

## Abstract

**Background:**

Bladder cancer is one of the most common urological cancers. Non-muscle invasive bladder cancer (NMIBC) accounts for about 75-85% of all newly diagnosed bladder cancers. Globally, there are many NMIBC-related publications. However, a bibliometric analysis of these publications has not been performed.

**Objective:**

This study aims to systematically analyze and visualize NMIBC-related publications through bibliometrics, and to reveal identified topics, hotspots, and knowledge gaps in related fields.

**Methods:**

Based on the Web of Science core collection database, we firstly analyzed the quantity and quality of publications in the field of NMIBC, secondly profiled the publishing groups in terms of country, institution, author’s publication and cooperation network, and finally sorted out and summarized the hot topics of research.

**Results:**

This bibliometric analysis was conducted from 2001 to 2022. The analysis identified 2,185 articles and reviews, which were published in 402 journals. The number of publications and citations on NMIBC-related research has steadily increased over the last two decades. Furthermore, academic institutions in Europe and the United States play a leading role in NMIBC research. The country, institution, journal, and author with the most publications were the United States (559), Radboud University Nijmegen (88), Urologic oncology: Seminars and Original Investigations (141), and Witjes J (74), respectively. The most frequently used keywords were Bladder cancer (793), Recurrence (671), Urothelial carcinoma (593), Progression (523), Bacillus-calmette-guerin (411), Transitional-cell carcinoma (401), Carcinoma (366), Risk (297), Transurethral resection (286), and Non-muscle-invasive bladder cancer (280).

**Conclusion:**

More and more scholars are devoted to the research of related NMIBC. This bibliometric analysis revealed that the main research topics and hotspots in NMIBC included pathological staging, clinical diagnosis and treatment, and bladder perfusion.

## Introduction

Bladder cancer is the most common malignancy of the urinary system. Its incidence is the first among malignancy of urinary system, and it is also the tenth most common cancer in the world (1). Approximate 550,000 people are diagnosed with bladder cancer each year, accounting for about 3.0% of all new cancer diagnoses. Furthermore, bladder cancer accounts for about 2.1% of all cancer deaths (2, 3). Incidence rates of Bladder cancer are consistently lower in women than men, the male-to-female incidence ratio is approximately 3:1 (4, 5). Bladder cancer is divided into two categories according to the depth of invasion. Non-muscle invasive bladder cancer (NMIBC) is regarded as the pathological stage below T2, while muscle-invasive bladder cancer (MIBC) falls under the pathological stage T2 and above. The NMIBC accounts for approximately 75-85% of new bladder cancer cases (6).

The pathogenesis of NMIBC is still unclear. However, bladder cancer is associated with various risk factors, including smoking, gender, occupation, and age (7). Currently, transurethral resection of bladder tumor (TURBT) combined with postoperative intravesical instillation is the treatment mainstay for NMIBC. However, this treatment method is associated with a high risk of intraoperative bleeding, bladder perforation, and induction of obturator nerve reflex (8). Bacillus Calmette-Guerin (BCG) immunotherapy has been used as an intravesical agent for decades to prevent the progression of high-risk NMIBC (9, 10). Despite its proven efficacy, there is no consensus on the optimal timing, dose, strain, and definition of BCG failure.

Bibliometrics is the quantitative analysis of literature, which is widely used for evaluating research trends and hotspots in various fields (11). VOSviewer and CiteSpace software are commonly used in bibliometrics for co-word analysis, co-citation analysis, and literature coupling analysis. These softwares can visually display the outcomes. They have the advantage of clustering technology and map presentation. They can rapidly analyze research trends in a certain field and exhibit them in the form of multivariate integrated visual knowledge maps (12, 13).

Hundreds of original articles and reviews on NMIBC are published each year. However, so far, there is no systematic analysis of NMIBC-related publications. Therefore, this study aimed to systematically analyze and visualize NMIBC publications over the past 20 years using related bibliometric softwares, such as VOSviewer and CiteSpace. Furthermore, the study aims to summarize the achievements attained in this field, understand the research direction and hotspot areas, and provide a reference for future studies.

## Methods

### Ethics statement

The present study did not involve any human subject participation, and it was entirely performed using the bibliometric data retrieved from the Web of Science database (WOS, https://www.webofscience.com/wos/woscc/basic-search) and hence, it is deemed to be exempted from the Institutional Review Board approval.

### Data sources and collection

The WOS database is the most commonly used and widely accepted database in scientific or bibliometric research. It contains nearly 9,000 high-impact journals and more than 12,000 academic conference proceedings, which provide a comprehensive overview of research in the scientific, technological, and medical research fields (14, 15).

Publications on NMIBC were retrieved on August 02, 2022. The time span was set between January 01, 2001, and August 01, 2022. First, the “WOS Core Collection” was selected on the search page. The search was refined to the subject headings “non-muscle invasive bladder cancer” and “bladder cancer” and article types “article” and “review”. “Plain text” was chosen for the file format, while “Full Record and Cited References” was chosen for the record content.

The search query string was described as follows: “non-muscle invasive bladder cancer” (Topic) AND “bladder cancer” (Topic) and Article or Review Article (Document Types) and Book Chapters (Exclude-Document Types).

### Data analysis

The data were downloaded and analyzed by two researchers respectively to assure the accuracy of data and the repeatability of the research. Microsoft Excel 2019 and GraphPad Prism 7 were applied to analyze the targeted files and exported the line charts and tables of top-cited or productive countries/regions, institutions, authors, journals, references, keywords.

The test of fit (R^2^) was used to predict the relationship between publication year and publication output to compare the degree of agreement between the predicted results and the actual occurrence. The closer R^2^ is to 1, the better the fitting degree of the regression line to the observed value is; otherwise, the worse it is. At the same time, the 2021 version of Impact factor (IF) and Journal impact factor quartile (JIF) quartile, as important indicators to measure the scientific value of research, were also included in the analysis.

### Bibliometric analysis and visualization software

CiteSpace (https://citespace.podia.com/download, R6.1.3) is a visual analysis software widely used in scientific papers. It is based on scientometrics data and information visualization technology. Further, it presents the knowledge structure by analyzing the underlying knowledge, patterns, and distribution of the literature (16). In this study, CiteSpace was used for keyword clustering and burst word analysis.

VOSviewer (https://www.vosviewer.com/, R1.6.18) is a bibliometric analysis software for mapping knowledge. It can be used for co-word analysis, co-citation analysis, coupling analysis of documents, and to visualize the results (17). In this study, VOSviewer was used to visualize countries, authors, institutional collaborations, cited journals, keyword co-occurrences and to construct density maps.

## Results

### Analysis of global publishing trends

**Figure 1:**
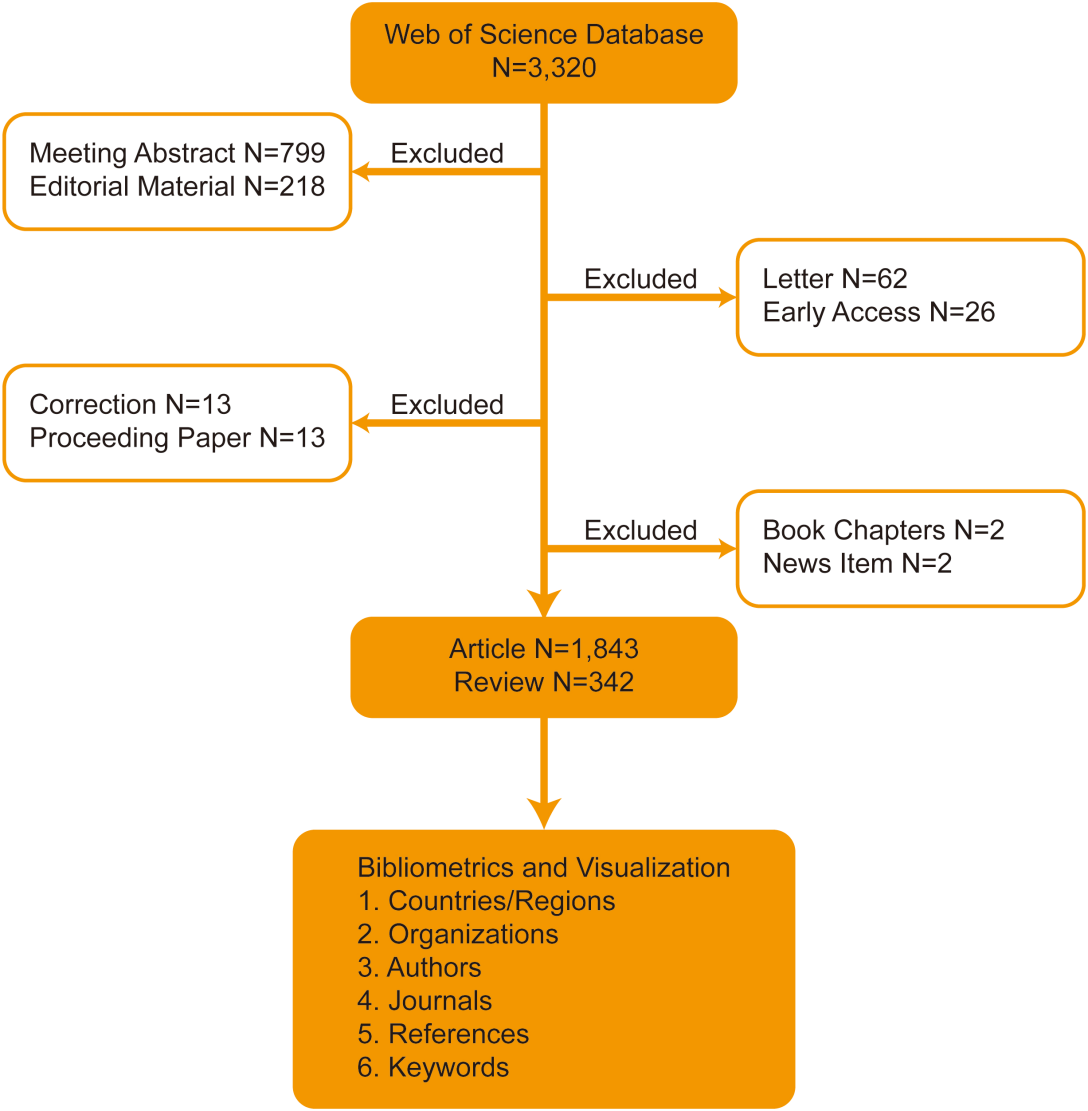
Flowchart of the search strategy.

Analysis of the number of published papers made annually is important as it reflects the growth of knowledge in a certain field. In this study, 2,185 articles and reviews on NMIBC research were selected out of the 3,320 records retrieved from the Web of Science database. A total of 1,135 conference abstracts, letters, news, and editorial materials were excluded (**Figure 1**).

**Figure 2:**
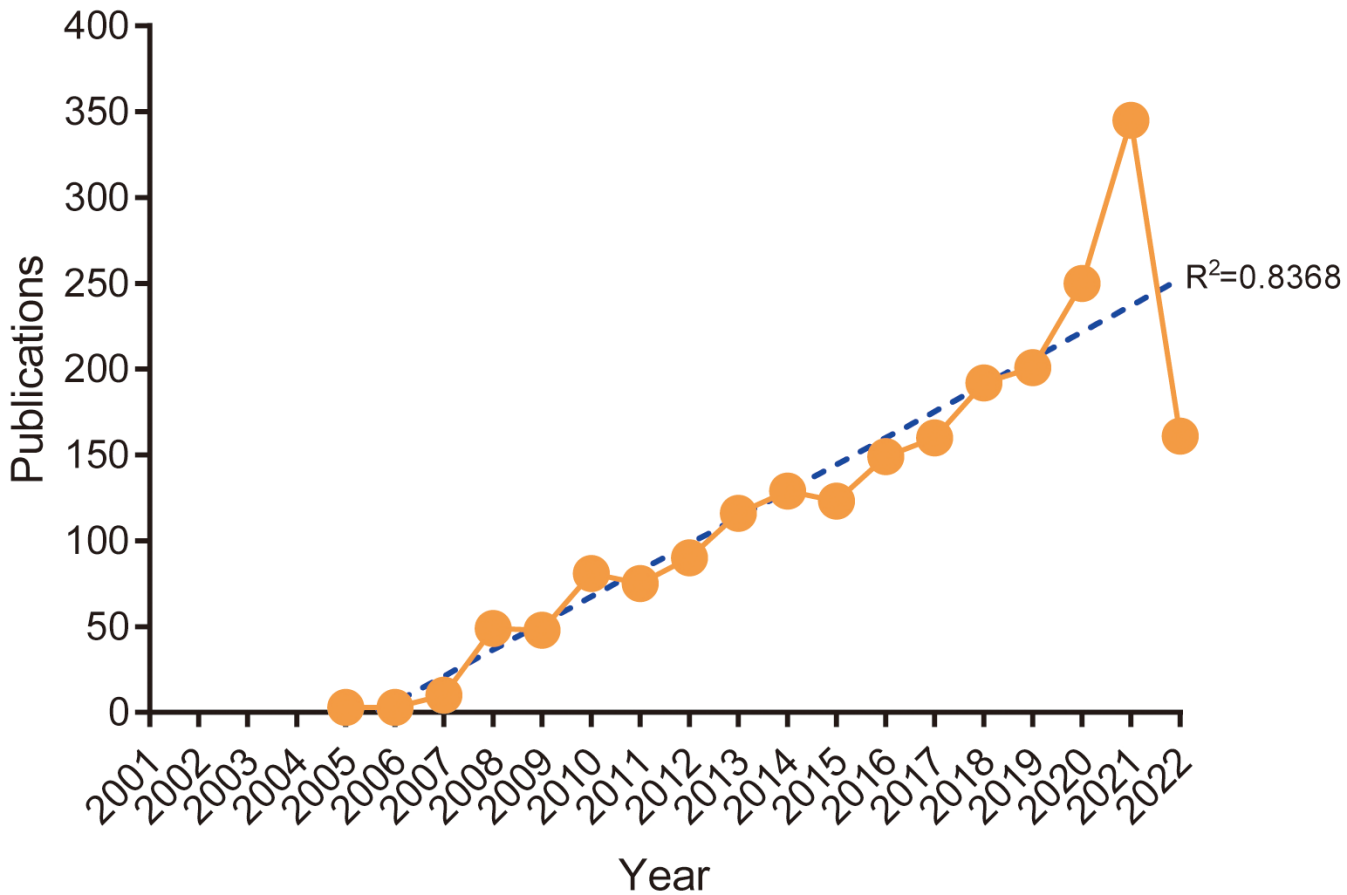
Annual trends of global publications.

Although the time span of our search was 2001-01-01 to 2022-08-01, no suitable papers were included before 2005. The reason is that in these 4 years, no Topic included both “non-muscle invasive bladder cancer” and “bladder cancer”, and conforms to “article” and “review” type papers. This study showed a slow growth of publications on NMIBC from 2005 to 2008. The first inflection point was noted in 2008, after which the number of published articles showed steady growth. The second inflection point was noted in 2018. However, after 2018, the number of NMIBC-related research papers significantly increased, with more than 200 papers being published annually to reach a record high in 2021. This bibliometric analysis generally showed a linear growth trend (R^2 =^ 0.8368) in NMIBC-related research, reflecting the increasing interest in this research field (**Figure 2**).

### Analysis of distribution and cooperation of leading countries/regions

**Figure 3:**
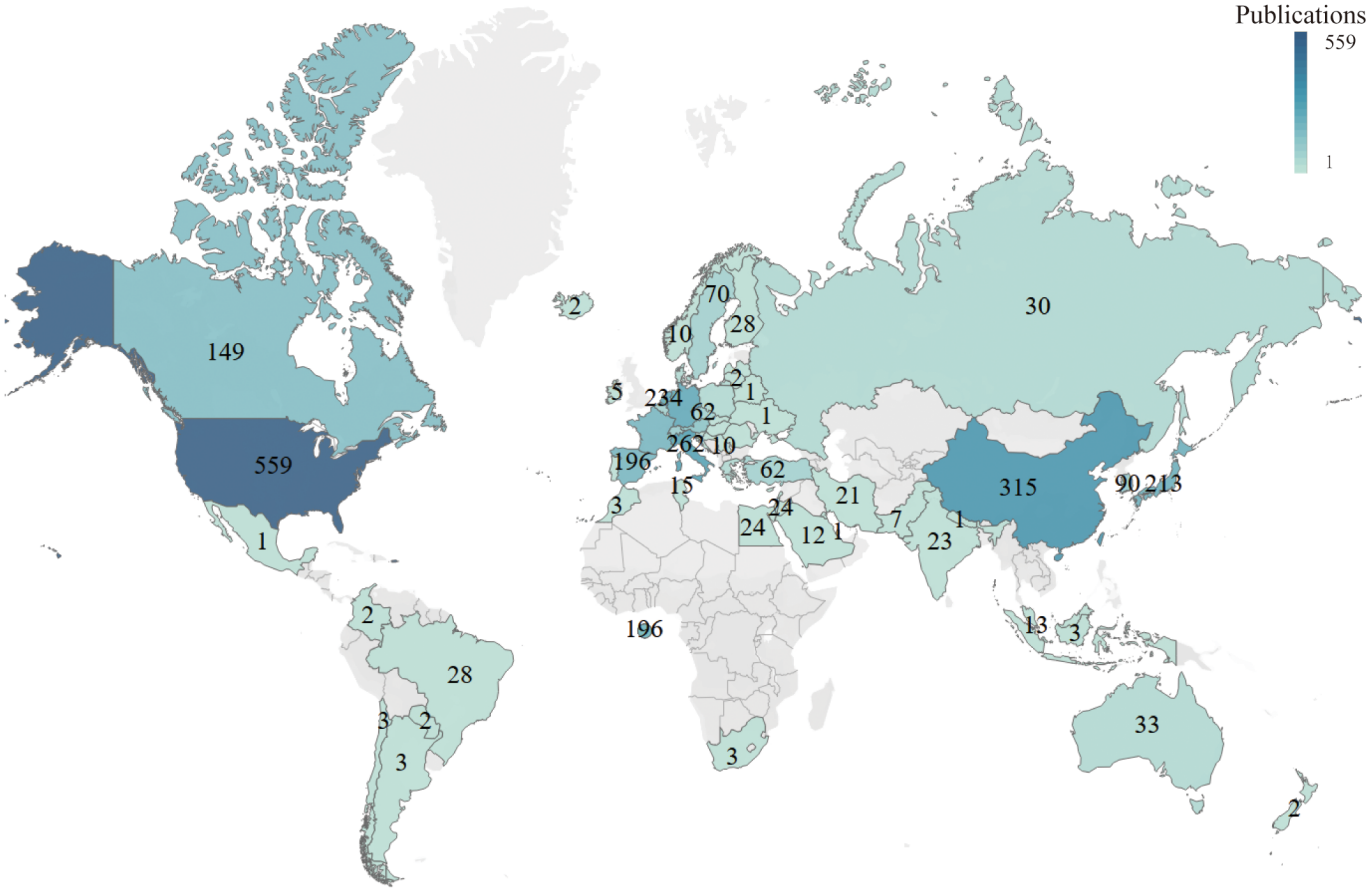
Global analysis of the research trends in NMIBC based on the origin of the publications.

A total of 69 countries/regions have published papers on NMIBC. The United States of America (USA) had the highest number of publications (559, 25.58%), followed by China (315, 14.42%), Italy (262, 11.99%), Germany (251, 11.49%), and the Netherlands (234, 10.71%) (**Table 1** and **Figure 3**). The highest number of total citations were from the USA (16,275), Germany (12,293), the Netherlands (12,173), Spain (10,221), and England (8,455) (**Table 1**). These results show that these countries are more interested in NMIBC-related research.

**Table 1:** Top 10 Countries/Regions by publications and citations.

Rank	Countries/Regions	Publications	% of 2,185	Rank	Countries/Regions	Citations
1	USA	559	25.58	1	USA	16,275
2	China	315	14.42	2	Germany	12,293
3	Italy	262	11.99	3	Netherlands	12,173
4	Germany	251	11.49	4	Spain	10,221
5	Netherlands	234	10.71	5	England	8,455
6	Japan	213	9.75	6	France	8,172
7	Spain	196	8.97	7	Italy	8,098
8	England	196	8.97	8	Belgium	7,962
9	France	182	8.33	9	Austria	6,399
10	Canada	149	6.82	10	Canada	6,302

**Figure 4:**
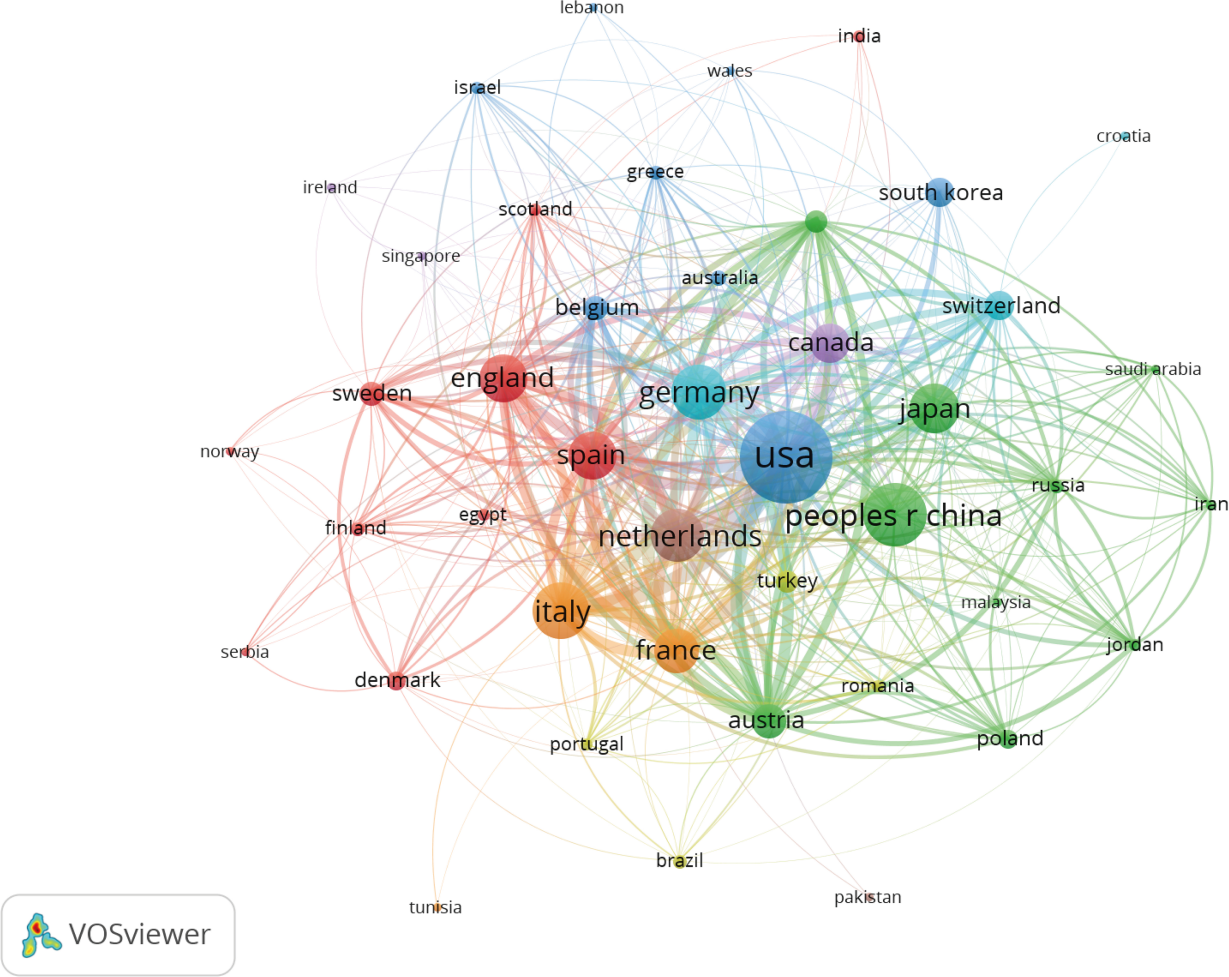
Co-occurrence map of Countries/Regions. The size of the nodes represents the number of articles; the thickness of the curve represents the strength of the collaboration; the colors represent different collaboration groups.

VOSviewer was used to analyze the cooperation of different countries. The line between nodes indicates the co-authorship between countries; the thicker the line, the stronger the cooperative relationship. The results showed that the USA, China, and Italy had more cooperation with other countries. However, cooperation between the other countries was weaker (**Figure 4**).

### Analysis of distribution and cooperation of leading institutions

**Figure 5:**
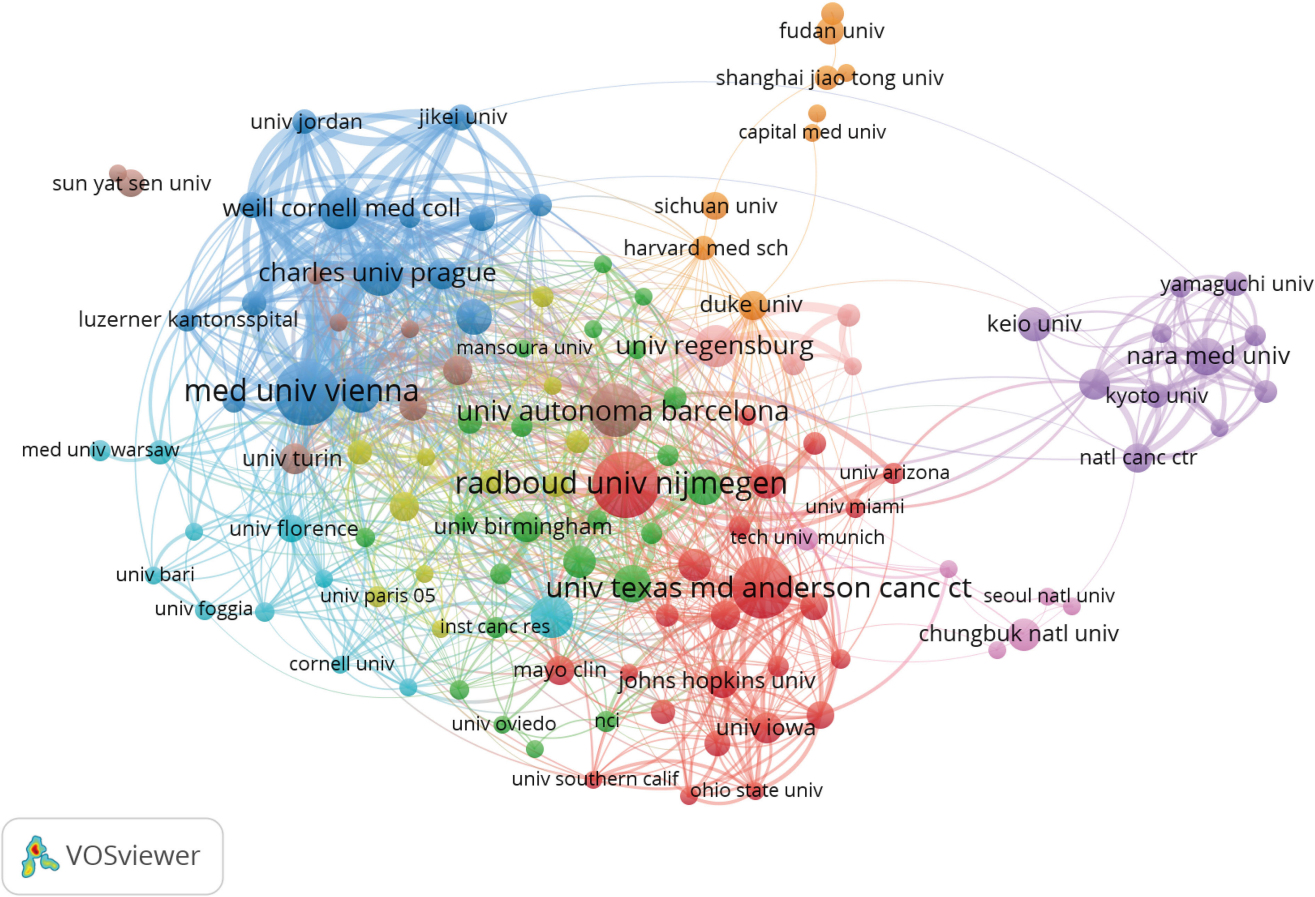
Co-occurrence map of Institutions. The size of the nodes represents the number of articles; the thickness of the curve represents the strength of the collaboration; the colors represent different collaboration groups.

A total of 2,978 institutions were involved in publishing NMIBC-related papers. The top five institutions with the highest number of publications were Radboud University Nijmegen (88), Medical University of Vienna (85), University of Texas MD Anderson Cancer Center (77), Autonomous University of Barcelona (63), and Charles University Prague (48) (**Table 2**). The top five institutions with the highest number of total citations were Autonomous University of Barcelona (6,122), Charles University Prague (5,390), Medical University of Vienna (5,159), Radboud University Nijmegen (4,052), and University of Regensburg (3,769) (**Table 2**). Radboud University Nijmegen and the University of Texas MD Anderson Cancer Center were at the center of the partnerships. However, most institutions were fragmented and lacked cooperation. The overall network density was low (density = 0.0175), mainly in the European and US institutions (**Figure 5**).

**Table 2:** Top 10 Institutions by publications and citations.

Rank	Organizations	Publications	Original country	Rank	Organizations	Citations	Original country
1	Radboud University Nijmegen	88	Netherlands	1	Autonomous University of Barcelona	6,122	Spain
2	Medical University of Vienna	85	Austria	2	Charles University Prague	5,390	Czech Republic
3	University of Texas MD Anderson Cancer Center	77	USA	3	Medical University of Vienna	5,159	Austria
4	Autonomous University of Barcelona	63	Spain	4	Radboud University Nijmegen	4,052	Netherlands
5	Charles University Prague	48	Czech Republic	5	Universität Regensburg	3,769	Germany
6	Regenburg University	43	Germany	6	Antoni Van Leeuwenhoek Hospital	3,235	Netherlands
7	Sloan Kettering Cancer Research Center	42	USA	7	Medical University of Graz	3,201	Austria
8	Weill Cornell Medical College	41	USA	8	EORTC Headquarters	3,065	Belgium
9	University of Texas Southwest Medicine	38	USA	9	Hyvinkaa Hospital	3,048	Finland
10	Nara Medical University	34	Japan	10	University of Texas MD Anderson Cancer Center	2,909	USA

### Analysis of authors and co-cited authors

**Figure 6:**
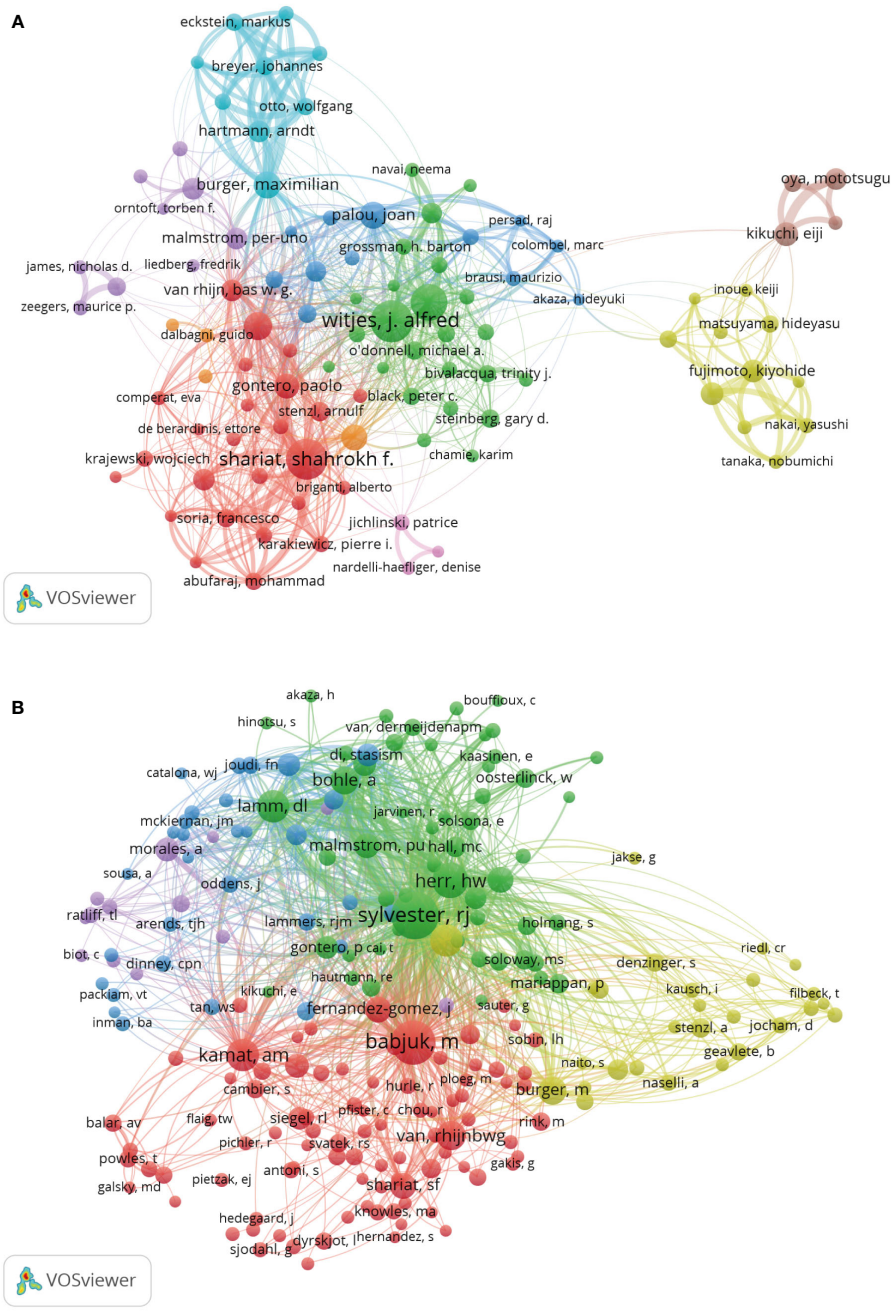
Analysis of Authors and Co-Cited Authors. **(A)** Co-occurrence map of Authors. The size of the nodes represents the number of articles. **(B)** Co-Cited Authors analysis map. The size of the nodes represents the number of co-citations.

The author co-occurrence analysis identified the core authors in NMIBC-related research and the strength of collaboration between authors. Co-cited analysis means that when two authors or papers are cited by a third author or paper at the same time, the two authors or papers have a co-cited relationship. This analysis revealed a total of 9,979 authors and 23,062 co-cited authors. Among them, Witjes J (74), Shariat S (70), Kamat A (58), Roupret M (40), and Burger M (37) had the highest publications (**Table 3** and **Figure 6A**). Furthermore, Witjes J and Shariat S showed the highest cooperation (**Figure 6A**). However, the collaboration of other authors and teams was weak, and the research was fragmented. The co-citation analysis showed that Sylvester R (1,651), Babjuk M (1,373), Herr H (799), Kamat A (614), and Witjes J (603) had the most co-citations (**Table 3** and **Figure 6B**). These results show that these authors are increasingly interested in NMIBC-related research.

**Table 3:** Top 10 Authors and Co-Cited Authors.

Rank	Authors	Publications	Rank	Authors	Citations	Rank	Co-Cited Authors	Co-Citations
1	Witjes J	74	1	Sylvester R	4,855	1	Sylvester R	1,651
2	Shariat S	70	2	Babjuk M	4,796	2	Babjuk M	1,373
3	Kamat A	58	3	Shariat S	4,765	3	Herr H	799
4	Roup R	40	4	Burger M	4,729	4	Kamat A	614
5	Palou J	37	5	Roup R	4,439	5	Witjes J	603
6	Burger M	37	6	Van Rhijn B	4,188	6	Lamm D	600
7	Lotan Y	34	7	Witjes J	3,942	7	Van Rhijn B	445
8	Gontero P	33	8	Boehle A	3,862	8	Bohle A	423
9	Kikuchi E	30	9	Kaasinen E	3,731	9	Burger M	408
10	Fujimoto K	28	10	Palou J	3,256	10	Shariat S	344

### Analysis of leading journals and co-cited journals

All the selected papers were published in 402 journals. The top five high-yield journals were Urologic oncology: Seminars and Original Investigations (141), BJU International (119), European Urology (100), World Journal of Urology (93), and International Journal of Urology (51). The most cited journals were European Urology (13,153), BJU International (2,899), Urologic oncology: Seminars and Original Investigations (1,957), World Journal of Urology (1,756), and Nature Reviews Urology (1,144) (**Table 4**).

**Table 4:** Top 10 Journals by publications and citations.

Rank	Journals	Publications	IF (2021)	JIF quartile
1	Urologiconcology-Seminars and Original Investigations	141	2.954	Q2
2	BJU International	119	5.969	Q1
3	European Urology	100	24.267	Q1
4	World Journal of Urology	93	3.661	Q1
5	International Journal of Urology	51	2.896	Q2
6	Plos One	42	3.24	Q2
7	Urologia Internationalis	41	1.934	Q3
8	European Urology Focus	34	5.952	Q1
9	Bladder Cancer	34	1.449	Q2
10	Frontiers in Oncology	33	5.738	Q2
Rank	Journals	Citations	IF (2021)	JIF quartile
1	European Urology	13,153	24.267	Q1
2	BJU International	2,899	5.969	Q1
3	Urologiconcology-Seminars and Original Investigations	1,957	2.954	Q2
4	World Journal of Urology	1,756	3.661	Q1
5	Nature Reviews Urology	1,144	16.43	Q1
6	Plos One	1,019	3.24	Q2
7	Clinical Cancer Research	856	13.801	Q1
8	International Journal of Urology	764	2.896	Q2
9	UROLOGY	719	2.633	Q3
10	Cancer	636	6.921	Q2

Analysis of the co-cited journals showed that 4,863 journals were co-cited. The top five co-cited journals were Urology Journal (10,502), European Urology (9,958), BJU International (3,235), UROLOGY (2,867), and Journal of Clinical Oncology (1,899) (**Table 5**). The top 5 cited and co-cited journals were mostly ranked into quartiles- Q1 and Q2, reflecting an outstanding academic performance in NMIBC research.

**Table 5:** Top 10 Co-Cited Journals.

Rank	Co-Cited Journals	Co-Citations	IF (2021)	JIF quartile
1	Urology Journal	10,502	1.555	Q4
2	European Urology	9,958	24.267	Q1
3	BJU International	3,235	5.969	Q1
4	UROLOGY	2,867	2.633	Q3
5	Journal of Clinical Oncology	1,899	50.717	Q1
6	Urologiconcology-Seminars and Original Investigations	1,661	2.954	Q2
7	World Journal of Urology	1,379	3.661	Q1
8	Cancer Research	1,351	13.312	Q1
9	Clinical Cancer Research	1,212	13.801	Q1
10	International Journal of Cancer	948	7.316	Q1

### Analysis of references co-occurrence and burst references

**Figure 7:**
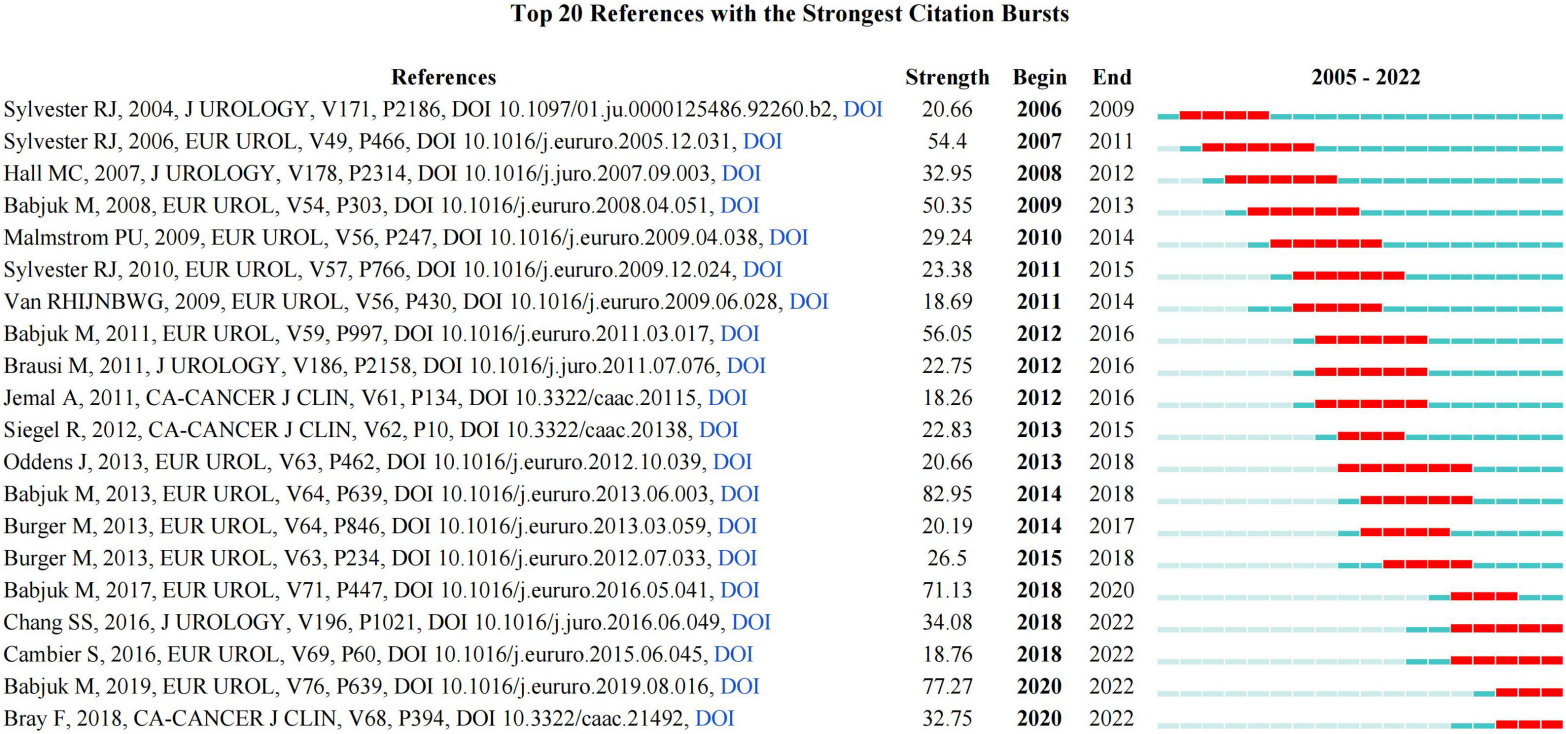
References burst analysis by CiteSpace.

A total of 2,185 references were obtained, of which seven references Babjuk et al., (18), Babjuk et al., (19), Kamat et al., (20) , Babjuk et al., (21), Chang et al., (22), Babjuk et al., (23), and Babjuk et al., (24) were cited more than 500 times (**Table 6**). In addition, a total of 20 references had the strongest citation bursts. The first reference that triggered a citation burst appeared in 2006 (Sylvester RJ, 2004). Most articles were cited between 2006 and 2018. The three references with the highest intensity were Babjuk et al., (19) (82.95), Babjuk et al., (23) (77.27), and Babjuk et al., (18) (71.13) (**Figure 7**).

**Table 6:** Top 10 References by citations.

Title	Journals	Authors	Year	Citations
EAU Guidelines on Non-Muscle-invasive Urothelial Carcinoma of the Bladder: Update 2016	European Urology	Babjuk M	2017	1,221
EAU Guidelines on Non-Muscle-invasive Urothelial Carcinoma of the Bladder: Update 2013	European Urology	Babjuk M	2013	919
Bladder cancer	Lancet	Kamat A	2016	642
EAU Guidelines on Non-Muscle-Invasive Urothelial Carcinoma of the Bladder, the 2011 Update	European Urology	Babjuk M	2011	615
Diagnosis and Treatment of Non-Muscle Invasive Bladder Cancer: AUA/SUO Guideline	Urology Journal	Chang S	2016	601
European Association of Urology Guidelines on Non-muscle-invasive Bladder Cancer (TaT1 and Carcinoma In Situ)-2019 Update	European Urology	Babjuk M	2019	588
EAU guidelines on non-muscle-invasive urothelial carcinoma of the bladder	European Urology	Babjuk M	2008	539
Recurrence and Progression of Disease in Non-Muscle-Invasive Bladder Cancer: From Epidemiology to Treatment Strategy	European Urology	van Rhijn B	2009	495
An Individual Patient Data Meta-Analysis of the Long-Term Outcome of Randomised Studies Comparing Intravesical Mitomycin C versus Bacillus Calmette-Guerin for Non-Muscle-Invasive Bladder Cancer	European Urology	Malmstrom P	2009	415
Bladder Cancer Incidence and Mortality: A Global Overview and Recent Trends	European Urology	Antoni S	2017	380

### Analysis of keyword co-occurrence, clustering and burst term

**Figure 8:**
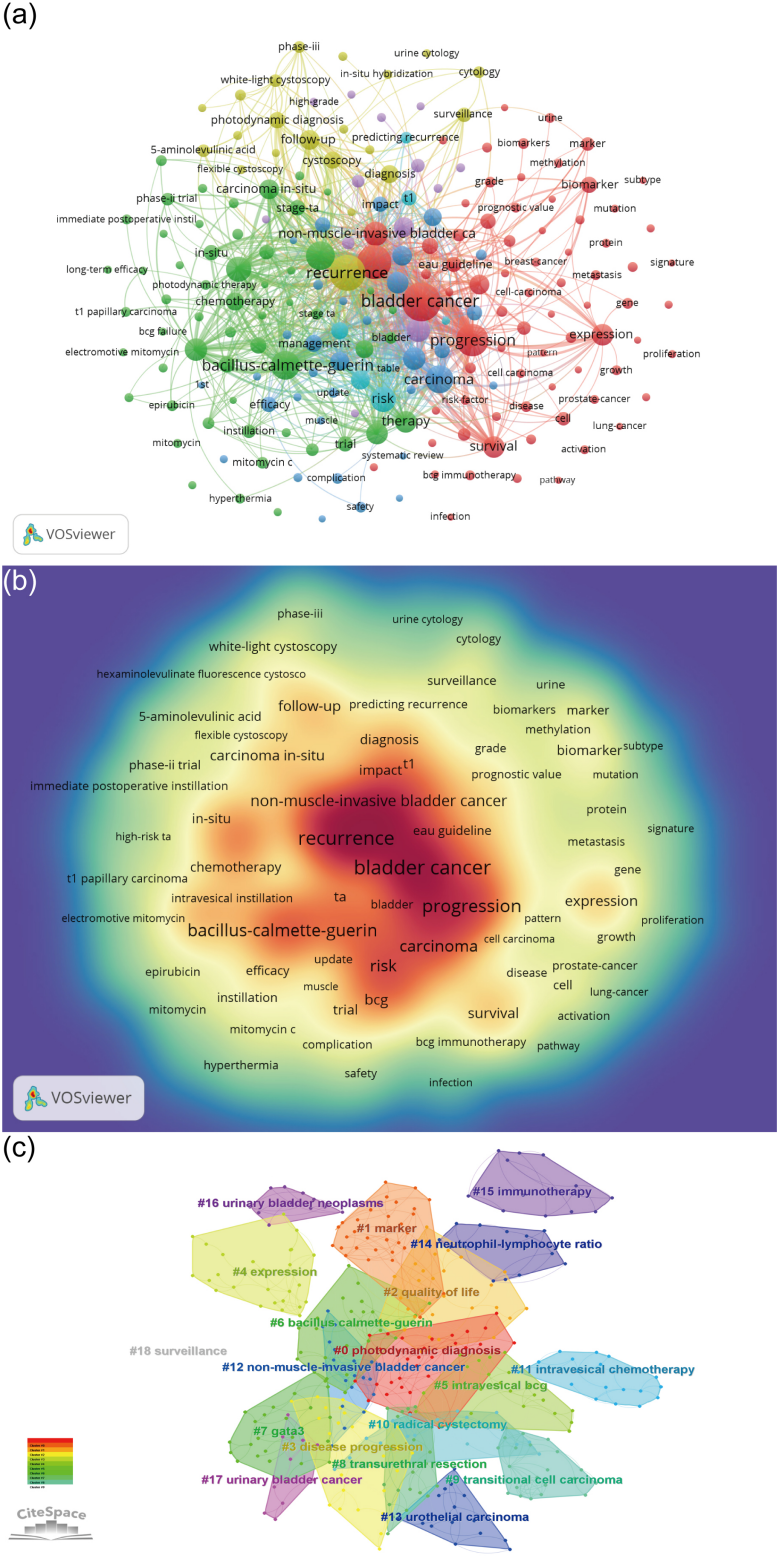
Keyword Analysis. **(A)** Keyword co-occurrence analysis map obtained using VOSviewer. The size of the nodes represents the number of occurrences; the thickness of the curve represents the strength of collaboration; the different colors represent the different clusters. **(B)** Keyword density visualization analysis. The higher the intensity of the red color node, the higher the number of keywords. **(C)** Keyword clustering map analysis through CiteSpace. A total of 19 categories of keywords were obtained. The different color blocks represent different keyword clusters.

Through keyword co-occurrence and burst analysis, we can identify the changing trend of research topics over time. A total of 5,422 keywords were obtained. The top ten keywords obtained were Bladder cancer (793), Recurrence (671), Urothelial carcinoma (593), Progression (523), Bacillus-calmette-guerin (411), Transitional-cell carcinoma (401), Carcinoma (366), Risk (297), Transurethral resection (286), and Non-muscle-invasive bladder cancer (280), **Figures 8A, B**. After clustering through CiteSpace software, a total of 19 clustering words were obtained, namely: #0 Photodynamic diagnosis, #1 Marker, #2 Quality of life, #3 Disease progression, #4 Expression, #5 Intravesical BCG, #6 Bacillus Calmette-Guerin, #7 Gata3, #8 Transurethral resection, #9 Transitional cell carcinoma, #10 Radical cystectomy, #11 Intravesical chemotherapy, #12 Non-muscle-invasive bladder cancer, #13 Urothelial carcinoma, #14 Neutrophil-lymphocyte ratio, #15 Immunotherapy, #16 Urinary bladder neoplasms, #17 Urinary bladder cancer, and #18 Surveillance, **Figure 8C**.

**Figure 9:**
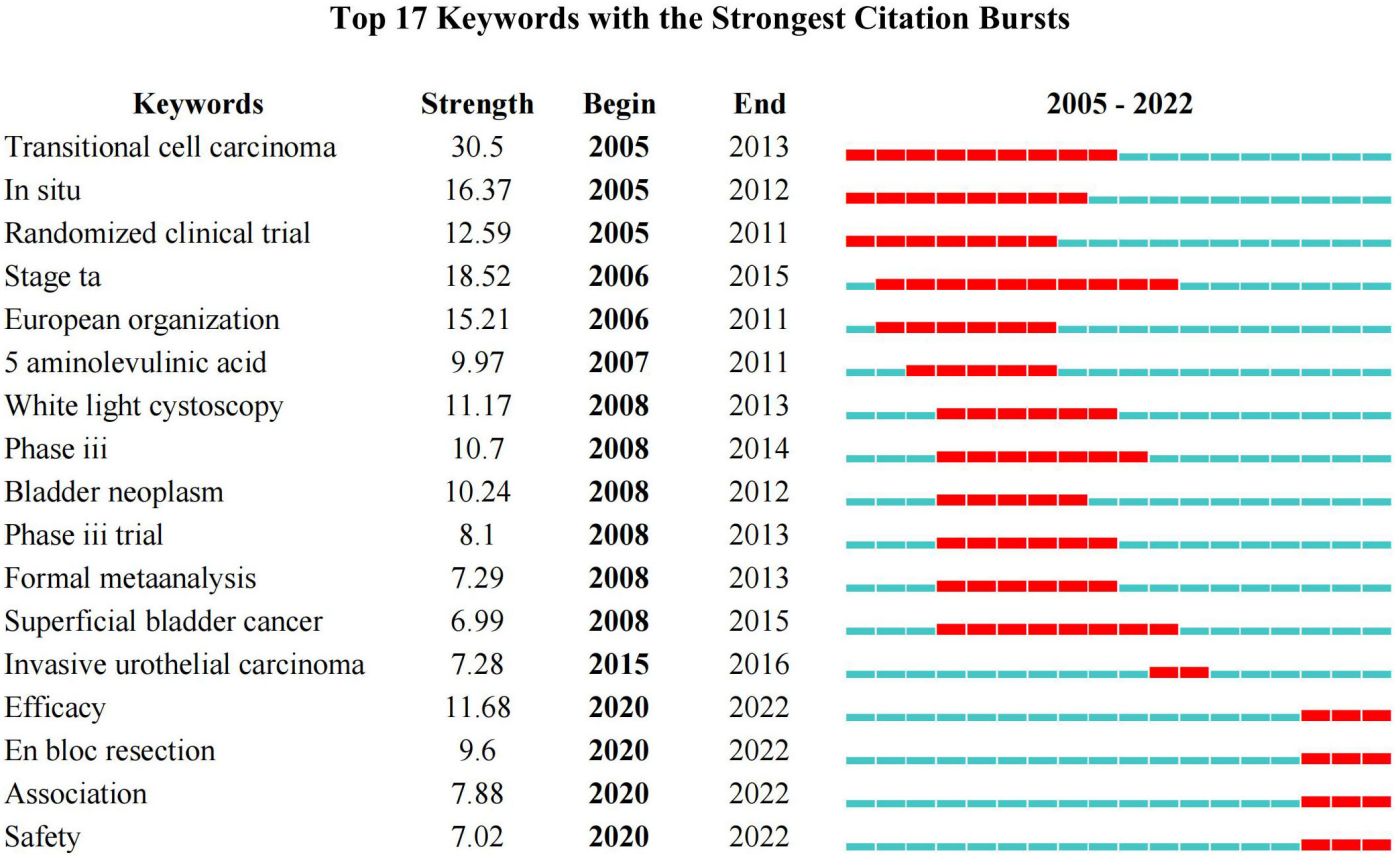
Keywords burst analysis by CiteSpace.

In addition, a total of 17 keyword-burst analysis results were obtained. From 2005 to 2015, the research hotspots in the NMIBC field were mainly randomized clinical trials and meta-analyses, which focused on cancer staging, and white light cystoscopy. However, from 2016 to 2022, the research hotspots in the NMIBC field are mainly focused on efficacy and safety. The three salient words with the highest strength were transitional cell carcinoma (30.5), stage Ta (18.52), and *in situ* (16.37), **Figure 9**.

## Discussion

With the advent of big data, researchers need to understand the developments within NMIBC-related research. The bibliometric analysis uses visualization softwares, such as VOSviewer and CiteSpace, to comprehensively analyze the existing literature, understand the research trends, and predict future research hotspots (25). This bibliometric analysisfocussed on NMIBC-related research in the past 20 years.

### General information on NMIBC-related literature

In the past 20 years, the number of published papers on NMIBC-related research showed a linear upward trend (R^2 =^ 0.8368).The highest publications were made in the past four years, with more than 200 publications being made each year. Most of the studies were funded by the National Science Foundation and multinational pharmaceutical companies, indicating that research in this field contributes to the national scientific and technological knowledge (26, 27). Most publications were made in the Urologic oncology: Seminars and Original Investigations journal, which was followed by BJU International, European Urology, World Journal of Urology, and International Journal of Urology, which are published in authoritative andrology and urology journals respectively. These journals have a significant impact on the NMIBC-related field. These journals have high impact factors and JIF quartiles, indicating that the papers published in these journals have high quality.

The US had the most publications and citations compared with other countries. However, although China ranked second in the number of published articles, it had a low number of citations ranking 14^th^. This finding shows that despite the yearly increase in publications in China, the published papers are not high quality. This could be attributed to the lack of international collaboration among researchers and language barriers. Among the top ten institutions with published papers, only Nara Medical University was from Asia. The rest were from Europe and the USA. Most institutions showed little cooperation. Therefore, there is a need to enhance communication and strengthen cooperation between research institutions and countries, especially in the Asian continent.

Witjes J had the highest publications. In addition, Sylvester R had the most co-citations, followed by Shariat S, Kamat A, Roupret M, Burger M, Herr H, and others. Witjes J mainly focused on reviews and systematic reviews with applications, such as hexaminolevulinate cystoscopy, BCG, and intravesical mitomycin C (28, 29). Sylvester R published five papers that had more than 500 citations (18, 19, 21, 23, 24), with the main contribution being the publication of NMIBC related guidelines. Among the seven references with more than 500 citations, five were related papers published by Babjuk and which also published on NMIBC-related guidelines (18, 19, 21, 23, 24). The other two highly cited references focused on the diagnosis, individualized treatment, and follow-up of NMIBC patients (20, 22). Sylvester R and Witjes J focused on NMIBC recurrence, progression, and BCG. Authors with the highest publications showed a solid cooperation relationship, suggesting that cooperative relationships are conducive to increasing the publishing of high-impact papers.

### Hotspots and Frontiers of NMIBC research

This bibliometric analysis revealed that the most influential references were mostly review articles and clinical guidelines from internationally renowned institutions and journals. The co-occurrence, clustering, and burst analysis of keywords identified pathological staging, clinical diagnosis and treatment, and bladder perfusion as the main topics and hotspots in the field of NMIBC research.

This study found that in the past 20 years, researchers have been more interested in the pathological staging of NMIBC. Further, the time span in the burst words was also longer, which could be because the different risk levels of NMIBC directly affect the recurrence and disease progression rates. Most guidelines were based on the 2009 American Joint Committee on Cancer/Union Internationale Contre le Cancer (AJCC/UICC) TNM staging system. These guidelines define NMIBC as noninvasive papillary UCB (Ta), carcinoma *in situ* (CIS) (Tis), or with limited invasion of the lamina propria (T1) (30). Furthermore, the guidelines define low-risk tumors as solitary, in stage Ta and without CIS and high-risk tumors as being multiple, in stage T1 and above, or with CIS. About 70% of newly diagnosed NMIBC cases present as stage Ta, 20% as stage T1, and 10% at stage CIS (31). At present, the main clinical problems are insufficient awareness of the risk of NMIBC, the low diagnosis rate of CIS, and inadequate treatment of high-risk NMIBC. Ritch et al. reported 5-year recurrence rates of 57%, 67%, and 77% and 5-year progression rates of 7%, 26%, and 46% for low-, intermediate-, and high-risk NMIBC, respectively (32).

The keyword analysis identified white light cystoscopy (WLC) and photodynamics as the main terms for examination and treatment modalities. The photodynamic-related keywords included Photodynamic Diagnosis (PDD), Photodynamic Therapy (PDT), and photosensitizer 5-aminolevulinic acid (5-ALA). The WLC-assisted endoscopy is currently the standard method for endoscopic detection and resection of bladder cancer (33). This method still has shortcomings, first, non-papillary bladder cancers, such as CIS and small tumors or concomitant tumors are difficult to visualize using WLC-assisted endoscopy (34). Second, WLC-assisted endoscopy may reveal a lower tumor grade than the actual grade, which could lead to incomplete resection of the tumor, thus increasing the likelihood of recurrence (35). These limitations have led to an increased interest in novel endoscopic imaging techniques, such as photodynamics. Photodynamic therapy was first applied for the diagnosis of bladder tumors in the 1970s. Photodynamic diagnosis can distinguish normal and cancerous tissues. 5-aminolevulinic acid (5-ALA) is a second-generation photosensitizer, which is advantageous due to lower photosensitivity toxicity, hypoallergenic reaction, and longer imaging time compared with the first-generation photosensitizer, Hematoporphyrinderivative (HPD) (36). 5-ALA-mediated PDD (5-ALA-PDD) is widely used in the diagnosis and treatment of NMIBC. Multiple randomized trials have shown that the diagnostic accuracy for 5-ALA-PDD in Ta, T1, and Tis stages of bladder cancer was 10% to 32% higher than that of WLC (37, 38). A recent randomized, double-blind, multicenter phase II/III clinical trial demonstrated the safety and sensitivity of oral 5-ALA in primary, recurrent bladder cancer, papilloma, and flat lesions. The sensitivity for oral 5-ALA-PDD was significantly higher than that of conventional white light cystoscopy (39). Other new endoscopic techniques, such as Raman spectroscopy, multiphoton microscopy, scanning fiber endoscopy, and molecular imaging, did not appear in the keyword analysis of this study due to their short development time and low citation rates.

Moreover, the keyword analysis identified intravesical instillation, especially with BCG, as one of the main categories. The treatment of NMIBC often combines TURBT with intravesical instillation to reduce the recurrence rate. Intravesical instillation is often carried out using chemotherapy drugs and immunotherapy agents. BCG is the main immunotherapy agent employed in intravesical instillation of NMIBC patients after surgery. In March 2020, the European Association of Urology (EAU) recommended that BCG treatment after TURBT was more effective than TURBT alone or TURBT plus intravesical chemotherapy in preventing tumor recurrence and halting disease progression (23). In the same year, the American Urological Association (AUA) and the Society of Urologic Oncology (SUO) issued similar recommendations. Furthermore, several studies have demonstrated that BCG infusion significantly reduces the long-term risk of recurrence and progression of NMIBC. Maintainance therapy with BCG for at least one year was more effective than chemotherapy in preventing recurrence in high-risk patients (40). However, studies have shown that patients treated with intravesical BCG have a higher incidence of local or systemic adverse events. In addition, approximately one-third of all patients treated with intravesical BCG show no response (41). Moreover, there are inconclusive findings on whether BCG affects cancer-specific mortality and the different strains of BCG to be employed in instillation therapy. Other chemotherapy drugs such as pirarubicin, epirubicin, gemcitabine, and interferon have questionable efficacy (42), which could be the reason why these drugs did not reflect in the keyword analysis of the present study.

Moreover, the keyword analysis revealed that research on NMIBC was more concentrated in the European region and institutions, probably due to the high incidence of NMIBC in developed countries, such as Europe and North America (43). Furthermore, more researchers from these regions pay more attention to NMIBC. A few studies focused on the pathological staging, clinical diagnosis, and treatment of NMIBC. Previous studies have shown that NMIBC is related to smoking, age, exposure to aromatic amines, aromatic polycyclic hydrocarbons, and chlorate hydrocarbons, or intake of water with high arsenic elements (44–46). However, the causative mechanisms of these factors are still unclear.

The significance of our research is as follows: 1. The countries/regions, institutions, authors, journals, references, keywords and other elements of NMIBC related literature in the past 20 years were clearly displayed. 2. Through the collation of data sets, readers and experts in relevant fields were helped to understand the development process, research status and knowledge hotspots of NMIBC. 3. Future research directions of NMIBC were further provided for reference. But, there are still some limitations to our study: 1. Recently published articles may have low citations due to the limited time available for citations, and thus the study may be prone to research bias. 2. This study only included articles and reviews published in English, which may have overlooked some of the literature. 3. With the rapid development of big data, this study may have a short timeframe and needs to be updated regularly.

## Conclusion

The number of published papers in the NMIBC field has been growing steadily since 2005, with over 200 papers published annually in the past three years. The USA and Europe are leading in research on NMIBC. However, there is a need to strengthen collaboration relationships, especially in developing countries. The top five cited and co-cited journals were mostly in Q1 and Q2, reflecting that research on NMIBC was of high quality. Furthermore, this bibliometric analysis revealed that the main research topics and hotspots in NMIBC included pathological staging, clinical diagnosis and treatment, and bladder perfusion.

## Data availability statement

The raw data supporting the conclusions of this article will be made available by the authors, without undue reservation.

## Author contributions

SD and JW designed the study. SD, ZY, and LX conducted the literature search. SD, FM, and LW analyzed the data and wrote the paper. JW and ZX approved the final manuscript. All authors contributed to the article and approved the submitted version.

## Funding

China Postdoctoral Innovative Talent Support Program (BX20220047); Young Talent Support Project of Beijing Association of Science and Technology (BYESS2022182); Young Talent Support Project of Chinese Association of Chinese Medicine (CACM-2021-QNRC2-B04).

## Conflict of interest

The authors declare that the research was conducted in the absence of any commercial or financial relationships that could be construed as a potential conflict of interest.

## Publisher’s note

All claims expressed in this article are solely those of the authors and do not necessarily represent those of their affiliated organizations, or those of the publisher, the editors and the reviewers. Any product that may be evaluated in this article, or claim that may be made by its manufacturer, is not guaranteed or endorsed by the publisher.
